# Supplementary material for: Exercise improves the outcome of anticancer treatment with ultrasound-hyperthermia-enhanced nanochemotherapy and autophagy inhibitor
Source: PLoS One. 2023 Jul 12;18(7):e0288380. doi: 10.1371/journal.pone.0288380 (PMC10337956; doi:10.1371/journal.pone.0288380)
Supplement: Attachment — Submitted filename: Response to Reviewers.docx [file pone.0288380.s002.docx]

Prof. E. Chenette

Editor-in-Chief, PLOS ONE

Dear Prof. Chenette,

The manuscript has been changed and modified point by point according to the comments and suggestions from the Reviewers. All authors have read and approved for submission of this manuscript to *PLOS ONE,* and any portion of the manuscript has not been appeared nor under consideration to other journals. We believe this combinatorial strategy significantly advances the knowledge for cancer treatment. The following is the response for the comments point by point:

**Editor’s comments:**

**Comment 1:** Please ensure that your manuscript meets PLOS ONE's style requirements, including those for file naming. The PLOS ONE style templates can be found at

<https://journals.plos.org/plosone/s/file?id=wjVg/PLOSOne_formatting_sample_main_body.pdf>

and

<https://journals.plos.org/plosone/s/file?id=ba62/PLOSOne_formatting_sample_title_authors_affiliations.pdf>

**Response 1:** The manuscript was carefully checked to meet PLOS ONE’s style requirements.

**Comment 2:** PLOS requires an ORCID iD for the corresponding author in Editorial Manager on papers submitted after December 6th, 2016. Please ensure that you have an ORCID iD and that it is validated in Editorial Manager. To do this, go to ‘Update my Information’ (in the upper left-hand corner of the main menu), and click on the Fetch/Validate link next to the ORCID field. This will take you to the ORCID site and allow you to create a new iD or authenticate a pre-existing iD in Editorial Manager. Please see the following video for instructions on linking an ORCID iD to your Editorial Manager account: <https://www.youtube.com/watch?v=_xcclfuvtxQ>

**Response 2:** The corresponding author, Prof. Win-Li Lin, has updated the information about ORCID iD in the Editorial Manager system.

**Comment 3:** In your Data Availability statement, you have not specified where the minimal data set underlying the results described in your manuscript can be found. PLOS defines a study's minimal data set as the underlying data used to reach the conclusions drawn in the manuscript and any additional data required to replicate the reported study findings in their entirety. All PLOS journals require that the minimal data set be made fully available. For more information about our data policy, please see http://journals.plos.org/plosone/s/data-availability.

"Upon re-submitting your revised manuscript, please upload your study’s minimal underlying data set as either Supporting Information files or to a stable, public repository and include the relevant URLs, DOIs, or accession numbers within your revised cover letter. For a list of acceptable repositories, please see http://journals.plos.org/plosone/s/data-availability#loc-recommended-repositories. Any potentially identifying patient information must be fully anonymized.

Important: If there are ethical or legal restrictions to sharing your data publicly, please explain these restrictions in detail. Please see our guidelines for more information on what we consider unacceptable restrictions to publicly sharing data: http://journals.plos.org/plosone/s/data-availability#loc-unacceptable-data-access-restrictions. Note that it is not acceptable for the authors to be the sole named individuals responsible for ensuring data access.

We will update your Data Availability statement to reflect the information you provide in your cover letter

**Response 3:** The underlying data set was uploaded as Supporting Information files.

**Reviewer’s comments:**

This is a potentially interesting study but it is limited by the small sample size and the conclusions made do not reflect the data.

SPECIFIC COMMENTS

**Comment 1:** Data availability. The authors state that “relevant data are within the manuscript and its Supporting Information files”. This is not the case; data availability refers to the anonymysed raw data, not the aggregated data reported in the manuscript. In line with PLOS ONE’s data availability policy (https://journals.plos.org/plosone/s/data-availability), please make the anonymised raw data available in the supplementary materials or within a public repository.

**Respnose 1:** The underlying data set was uploaded as Supporting Information files.

**Comment 2:** Please be specific in your title – exercise improved survival of anticancer treatment but it did not reduce tumour growth.

**Comment 3:** ABSTRACT - Line 40: Exercise did not reduce tumour growth. Please remove “but the difference was not significant” and make it clear in the conclusions that there was NO EVIDENCE that exercise reduced tumour growth in this experiment.

**Response 2&3:** Preclinical cancer studies often use in vivo animal models to assess the efficacy of anticancer treatment. A common study design is to repeatedly measure the volume of tumors over a period of time (longitudinal study) and analyze the difference at arbitrary time point (cross-sectional study), while the survival of animals is also recorded (survival study).

For animal welfare, there is usually a pre-defined tumor burden limit in *in vivo* study. However, the tumor burden limit may cause problems in data analysis. The animal bearing the largest tumor that exceeds the limit is removed from study first, reducing the group average size. The tumor size in the control group is disproportionally underestimated, causing biased estimate of treatment effect since the tumor shrinkage owing to treatment looks less significant [1]. Besides, the statistical power is reduced because the sample size decreased over time [1-2]. These factors may mask the true efficacy of treatment [1, 3].

That is what exactly happened in our study. To demonstrate such underestimation caused by dropout, we estimate the unobserved tumor volumes as if they had not been removed due to tumor burden limit. The tumor growth curves were transformed into logarithmic scale, and the unobserved values were linearly extrapolated from the last five observations, then the values were transformed back to ordinary scale.

The tumor growth curves with extrapolated values were plotted as following Figure s1. For simplicity, the control group was omitted in Figure s1. As a comparison, the Figure 2 in the original manuscript was placed together.

**Figure s1.** Tumor growth curves with estimate by extrapolation.

**Figure s2.**Tumor growth curves based on observed values. (from the original manuscript)

From these figures, it can be seen that the tumor volumes in PLD+pUH+CQ group were highly underestimated due to dropout. When we put those unobserved values back as if they had not been restricted by tumor burden limit, we derived a much larger estimate of tumor volumes in the PLD+pUH+CQ group. The underestimation of tumor volumes in the PLD+pUH+CQ group resulted in insignificant difference in cross-sectional comparison between HIIT+PLD+pUH+CQ group and led to biased estimate about the true efficacy of exercise. It also answers the question raise in our study: if exercise was ineffective in reducing tumor growth, how could it significantly prolong survival? The answer is that exercise could be actually effective but the analysis was biased.

Several approaches have been developed to address this problem. Many of them were based on advanced statistical techniques, such as ECM algorithm [4] or joint modelling of longitudinal and survival data [5]. Here we adopted the linear regression modelling method proposed by Zavrakidis et al. [3] to analyze the anticancer efficacy of exercise in our study.

Step 1, transform the tumor volumes over time into logarithm. Step 2, model the log-transformed tumor volumes with linear regression, with time and treatment as explanatory variables. Step 3, interpret the results, especially the coefficient corresponding to treatment.

Since we were interested in the role of exercise, the analysis compared the data from two groups: HIIT+PLD+pUH+CQ and PLD+pUH+CQ. The results of linear regression were summarized in the following Table 1.

**Table 1.** Summary of linear regression model of log-transformed tumor volumes

| **Linear regression** | | | | | |
| --- | --- | --- | --- | --- | --- |
| HIIT+PLD+pUH+CQ group versus PLD+pUH+CQ group | | | | | |
| Explanatory variables | Coefficient | Standard error | | *t* statistics | *p* value |
| Time | 0.064681 | 0.002634 | | 24.559 | < 2*10^-16^ ^***^ |
| Treatment | -0.196684 | 0.074287 | | -2.648 | 0.00888 ^**^ |
| Note. *** p<0.001, ** p<0.01, * p<0.05 | | | | | |
|  | | | | | |
| Multiple R-squared: 0.7832, | | | Adjusted R-squared: 0.7806 | | |
| F-statistic: 301.7 on 2 and 167 DF, | | | *p*-value: < 2.2*10^-16^ | | |

The F-statistic and corresponding p values along with R-squared values showed that the model provided valid and good linear fit to the log-transformed tumor volumes.

The p value for treatment was 0.00888, indicating that the tumor growth profiles of HIIT+PLD+pUH+CQ group and PLD+pUH+CQ group significantly differed.

The log-transformed tumor growth profiles were visualized as scatter plot with fitted line as following Figure s2.

Figure s2. Log-transformed tumor growth profiles with fitted line

It could be clearly seen that the fitted line of two groups differed in y-intercepts, and the difference was statistically significant. It meant that exercise could further reduce the tumor volume in the presence of PLD+pUH+CQ treatment.

We believed that above analysis showing significant difference between the two groups provided solid evidence to support our conclusion that exercise hampered tumor growth and improved the anti-tumor efficacy of treatment.

The whole manuscript was revised to include the linear regression analysis and results, as detailed below.

Title: unchanged.

Abstract, Line 40-41: Results showed that HIIT+PLD+pUH+CQ significantly reduced the tumor volumes and brought about longer survival of tumor-bearing mice than PLD+pUH+CQ did.

Materials and Methods, Statistical analysis, Line 226-232: The tumor growth curves were modelled with linear regression on log-transformed tumor volumes, and then the regression coefficients were analyzed. The survival of mice was analyzed with Log Rank (Mantel-Cox) test. Statistical significance was defined as p<0.05. Analysis of tumor growth curves were performed with StatsNotebook, an open-source statistical package built on R. Survival analysis was performed with IBM SPSS Statistics version 20.0 (IBM Corp., Armonk, NY, USA).

Results, the first subheading, Line 247-258: Fig. 2(a) showed the log-transformed growth curves of tumors under different treatment condition, and Fig 2(b) showed the fitted line with linear regression model. Mice experiencing 6 weeks of HIIT prior to tumor implantation and treatment had smaller subcutaneous 4T1-luc2 tumors when compared to the mice treated with PLD+pUH+CQ but without exercise, as demonstrated in Fig 2(b). The analysis of linear regression model was summarized in Table I. The F-statistics and R-squared values showed that the model provided valid and good linear fit to the log-transformed tumor volumes. The p value corresponding to treatment was 0.00888, indicating that there was significant difference in tumor growth curves between HIIT+PLD+pUH+CQ group and PLD+pUH+CQ group. These findings suggested that the addition of exercise into combination treatment strengthened the anti-tumor efficacy of PLD+pUH+CQ treatment.

Figure 2 was re-drawn and re-organized. The caption was revised accordingly.

Table I was added.

Discussion, the first paragraph, Line 325-326: We found that the tumor volume was significantly reduced by HIIT+PLD+pUH+CQ treatment than those treated with PLD+pUH+CQ.

Discussion, the first paragraph, Line 330-334: Furthermore, the survival time of tumor-bearing mice in the HIIT+PLD+pUH+CQ group was significantly longer than that in the PLD+pUH+CQ group. Exercise may improve the efficacy of anticancer treatment via multi-faceted effects, including modulating immunity and physiological changes.

Conclusion, Line 369-371: It was shown that mice receiving 6 weeks of high-intensity exercise before 4T1 tumor implantation had significantly smaller tumors and significantly longer survival after treatment than those received the same treatment without exercise.

[1] Martin EC, Aarons L, Yates JW. Accounting for dropout in xenografted tumour efficacy studies: integrated endpoint analysis, reduced bias and better use of animals. Cancer Chemother Pharmacol. 2016 Jul;78(1):131-41.

[2] Oberg AL, Heinzen EP, Hou X, Al Hilli MM, Hurley RM, Wahner Hendrickson AE, Goergen KM, Larson MC, Becker MA, Eckel-Passow JE, Maurer MJ, Kaufmann SH, Haluska P, Weroha SJ. Statistical analysis of comparative tumor growth repeated measures experiments in the ovarian cancer patient derived xenograft (PDX) setting. Sci Rep. 2021 Apr 13;11(1):8076.

[3] Zavrakidis I, Jóźwiak K, Hauptmann M. Statistical analysis of longitudinal data on tumour growth in mice experiments. Sci Rep. 2020 Jun 4;10(1):9143.

[4] Tan M, Fang HB, Tian GL, Houghton PJ. Repeated-measures models with constrained parameters for incomplete data in tumour xenograft experiments. Stat Med. 2005 Jan 15;24(1):109-19.

[5] Pan J, Bao Y, Dai H, Fang HB. Joint longitudinal and survival-cure models in tumour xenograft experiments. Stat Med. 2014 Aug 15;33(18):3229-40.

**Comment 4:** INTRODUCTION - Lines 48-49: The hallmarks of cancer were updated in 2022. Please revised accordingly. <https://aacrjournals.org/cancerdiscovery/article/12/1/31/675608/Hallmarks-of-Cancer-New-DimensionsHallmarks-of>

**Response 4:** The reference [1] was updated to the latest version according to the reviewer’s suggestion, and the descriptions in manuscript was revised to reflect the change as follows.

Line 48-50: The concept of “hallmarks of cancer”, first proposed by D. Hanahan and R.A. Weinberg in 2000 and updated in 2011 and 2022, described the complex and bizarre nature possessed by most types of cancer [1].

**Comment 5:** Lines 58-67: Most of the research between exercise and cancer outcomes (particularly the epidemiological evidence) relates to physical activity, not exercise per se. Exercise is a subcomponent of physical activity; it is planned, structured bouts of physical activity purposefully carried out to improve or maintain health or fitness. Please make this distinction before proceeding to discuss the impact of exercise on chemotherapeutic-related symptoms etc.

**Response 5:** We acknowledged the distinction between physical activity and exercise. Most of the literatures reviewed here (reference [9-19]) described studies that prescribed specific exercise interventions on specific groups of patients. We believed that the usage of “exercise” here was appropriate, so we kept the usage as the original authors used in their articles.

Instead, several observational studies cited in the subsequent paragraph (reference [20-22]) exactly discussed how physical activity influenced the prognosis of cancer. We revised the usage in this place to accurately reflect the concept in the cited literatures as follows.

Line 69-71: Several observational studies revealed that physical activity effectively reduced the recurrence of several types of cancer, including colorectal cancer, breast cancer, and prostate cancer [20-22].

**Comment 6:** Line 68: Change “researches” to “research studies”

**Response 6**: The sentence was revised according to reviewer’s suggestion.

Line 68-69: Besides functional improvement, there are a growing number of research studies indicating that exercise may also directly affect cancer cells.

**Comment 7:** Line 76: When discussing the role of interleukins in the exercise-induced regulation of NK cell mobilisation and cancer cell proliferation, it is worth acknowledging this recent study which highlighted a more direct role of IL-6: <https://onlinelibrary.wiley.com/doi/10.1002/ijc.33982>

**Response 7:** The mentioned literature was added as reference [28] and properly cited in the manuscript as follows.

Line 77-79: It was also reported that exercise-induced IL-6 inhibited the proliferation of malignant cells through regulation of DNA damage [28].

**Comment 8:** Line 85: What is meant by “works fine”? Please be more specific

**Response 8:** The sentence was revised to elaborate more clearly and precisely as follows.

Line 87-89: Hyperthermia as an adjunctive therapy with standard anticancer treatment such as chemotherapy or radiotherapy effectively improved the response rate and survival [40, 41].

**Comment 9:** Lines 104-109: Please mention the primary outcome(s) when discussing the aims of the study. For example, to be more specific, you might say “reduced tumour growth and recurrence” rather than “improve the outcome of treatment”.

**Response 9:** The paragraph was revised to describe the primary outcome of study more specifically as follows.

Line 107-113: The aim of present study is to utilize the multi-faceted effect of exercise prior to hyperthermia-facilitated nano-chemotherapy and autophagy inhibitor, in hope of a comprehensive and effective treatment strategy. We let mice do high-intensity interval training (HIIT) before they were implanted with 4T1 murine breast cancer cells. We treated the subcutaneous 4T1 tumor with combinatorial strategy with PLD, pUH, and CQ, to see if prior high-intensity exercise hindered tumor growth and prolonged the survival of tumor-bearing mice.

**Comment 10:** Lines 104-109: It should be made clear in your aims that you are investigating the effect of exercise BEFORE treatment. Please amend throughout.

**Response 10:** The manuscript was revised to more clearly describe that the aim of our study was to investigate the effect of exercise before treatment as follows.

Line 107-113: The aim of present study is to utilize the multi-faceted effect of exercise prior to hyperthermia-facilitated nano-chemotherapy and autophagy inhibitor, in hope of a comprehensive and effective treatment strategy. We let mice do high-intensity interval training (HIIT) before they were implanted with 4T1 murine breast cancer cells. We treated the subcutaneous 4T1 tumor with combinatorial strategy with PLD, pUH, and CQ, to see if prior high-intensity exercise hindered tumor growth and prolonged the survival of tumor-bearing mice.

**Comment 11:** Did you prospectively register the study on a publicly available database? If you did, please cite the registration. If not, please clearly state that this study was not prospectively registered.

**Response 11:** No, we did not prospectively register the study protocols. A clear statement about prospective registration was added into the Experimental design subheading in the manuscript as follows.

Line 121: The study protocol was not prospectively registered.

**Comment 12:** Please provide a justification for your sample size. Here is a good paper for reference: <https://online.ucpress.edu/collabra/article/8/1/33267/120491/Sample-Size-Justification>

**Response 12:** A statement justifying the sample size of study was added as follows.

Line 123-124: The number of animals was determined by the capacity of experiment instruments and operators.

**Comment 13:** Please include a subheading that clearly details the outcomes of the study. This information seems to be under the “study design” subheading at the moment, which is not appropriate. In this section, please detail how recurrence was operationalised. Please also include a description of the methods used to analyse blood cell compartments.

**Response 13**: A subheading that detailed the **outcomes** including the **criteria of recurrence** was added into the Methods section, and the methods of **blood cell components** analysis was added in another subheading as follows.

Line 138-158:

Outcomes of anti-tumor efficacy study

The principal outcome indicators for anti-tumor efficacy were the tumor growth response over time and the survival of tumor-bearing mice. The volumes of subcutaneous tumors were measured with digital caliper every three to four days and their volumes were calculated as 0.5*length*width*width. The time of recurrence was defined when the tumor growth curve has passed treatment-induced nadir and the tumor regrow to an extent exceeding its pre-nadir peak volume.

Survival was determined by disease-related death, tumor volume exceeding the pre-defined tumor burden limit, or moribundity. In accordance with animal welfare guidelines, when the volume of subcutaneous tumor exceeded 1000 mm3, the tumor-bearing mouse was considered reaching humane endpoint and was euthanized immediately after tumor volume measurement by cervical dislocation under isoflurane anesthesia. In addition to tumor volume, the body weight and health condition of mice were observed every day for clinical signs indicating severe discomfort due to growing cancer. In case of mice expressed signs for moribundity such as rapid decline in body weight (>20%), decreased appetite, or hunched posture, the mice were euthanized to end its suffering. The implanted tumors were also closely monitored for signs such as ulceration or bleeding. In case mice had tumors with unhealed ulcers or active bleeding and showed signs of severe discomfort, they were considered as reaching early endpoint and were euthanized for the sake of humane consideration. No mice died before meeting criteria of euthanasia.

Line 213-222:

Blood cell components analysis

Blood cell components pre-exercise and post-exercise were analyzed to understand the change in physiological environment induced by exercise. Four mice began six weeks of HIIT when they were 7-week-old. The details of HIIT were described in Exercise treatment section. The blood cell components data was collected before they started HIIT as a baseline (7-week-old) and was collected again after they completed six-week training course (13-week-old). Another six mice of the same age as the four mice receiving HIIT were used to control the effect of aging. They received no specific treatment, and the blood cell components were collected when they were 7-week-old and 13-week-old, respectively.

**Comment 14:** Line 181: Please provide a reference to support your statement that 74.2% VO2max exceeds the threshold of high-intensity training.

**Response 14:** The related literature was added as reference [56] and properly cited in the manuscript as follows.

Line 191-195: Based on the relationship between running speed and maximal oxygen consumption (VO_2_max) in mice proposed by Fernand et al. [55], such running speed corresponds to 74.2% VO_2_max. It is equivalent to 82.5% HRmax according to the equation developed by Lounana et al. [56], exceeding the threshold of high intensity training.

**Comment 15:** Lines 175-184: Did all mice in the exercise condition complete every single planned bout of exercise?

**Response 15:** Yes, all mice in the exercise group completed every planned bout of exercise.

**Comment 16:** Line 201: Tumour volume was measured every 3 days – this is a huge number of comparisons. Did you correct for multiple comparisons in your analysis? You should have done because you employed frequentist statistics, and frequentist statistics is grounded in controlling type I error rate. The results may need to be revised to incorporate error control.

**Response 16:** We changed the method to analyze tumor growth profile in the revised manuscript.

In the original manuscript, we measured tumor volumes at multiple time points and performed cross-sectional comparisons between groups. This approach was prone to accumulated type I error. Usually a correction for multiple comparison is required, but most correction methods assume independencies among comparisons. Such assumption is highly unlikely in tumor growth study, causing great challenge in the correction of multiple comparison.

Instead, we chose the linear regression modelling methods to analyze tumor growth profiles. The multiple comparison problem was avoided since the number of comparisons involved in our regression model was much less.

Besides, the linear regression modelling method also possessed several advantages over the method in our original manuscript. First, it considers the whole shape of tumor growth curve, whereas cross-sectional comparison method only considers the information at certain time point. Second, linear regression modelling is less affected by dropout due to tumor burden limit, which may cause biased estimate in cross-sectional comparisons.

First, we transformed the tumor growth profiles into logarithm, then model the log-transformed tumor volumes with linear regression, with time and treatment as explanatory variables. Since we were interested in the role of exercise, the analysis compared the data from two groups: HIIT+PLD+pUH+CQ and PLD+pUH+CQ. The results of linear regression were summarized in the following Table 1.

**Table 1.** Summary of linear regression model of log-transformed tumor volumes

| **Linear regression** | | | | | |
| --- | --- | --- | --- | --- | --- |
| HIIT+PLD+pUH+CQ group versus PLD+pUH+CQ group | | | | | |
| Explanatory variables | Coefficient | Standard error | | *t* statistics | *p* value |
| Time | 0.064681 | 0.002634 | | 24.559 | < 2*10^-16^ *** |
| Treatment | -0.196684 | 0.074287 | | -2.648 | 0.00888 ** |
| Note. *** p<0.001, ** p<0.01, * p<0.05 | | | | | |
|  | | | | | |
| Multiple R-squared: 0.7832, | | | Adjusted R-squared: 0.7806 | | |
| F-statistic: 301.7 on 2 and 167 DF, | | | *p*-value: < 2.2*10^-16^ | | |

The F-statistic and corresponding p values along with R-squared values showed that the model provided valid and good linear fit to the log-transformed tumor volumes.

The p value for treatment was 0.00888, indicating that the tumor growth profiles of HIIT+PLD+pUH+CQ group and PLD+pUH+CQ group significant differed.

**Comment 17:** Lines 224-226: This statement is incorrect; your study provided NO EVIDENCE that exercise strengthened the anti-tumour efficacy of treatment because there were no sig differences. Please revise accordingly.

**Response 17: (as Comment 2&3)**

**Comment 18:** Figures 4-6 – it is best practise to plot individual data points when you have small sample sizes (https://pubmed.ncbi.nlm.nih.gov/31608303/). Thus, please re-draw the graphs using individual data points.

**Response 18**: Figure 4-6 were re-drawn to simultaneously represent individual data points along with their average, and the captions were revised accordingly as follows.

**Fig. 4.** The percentage of neutrophils among white blood cells (NEUT%). Green symbols indicated the values of NEUT% of individual mice in the exercise group obtained before and after 6 weeks of high-intensity exercise. Blue symbols were the values of NEUT% of individual mice in the no exercise group obtained at the same time points for comparison. * p<0.05.

**Fig. 5.** The percentage of lymphocytes among white blood cells (LYMPH%). Green symbols indicated the values of LYMPH% of individual mice in the exercise group obtained before and after 6 weeks of high-intensity exercise. Blue symbols were the values of LYMPH% of individual mice in the no exercise group obtained at the same time points for comparison. * p<0.05.

**Fig 6.** (a) The number of reticulocytes (RET) and (b) the percentage among red blood cells (RET%). Green symbols indicated the values of individual mice in the exercise group obtained before and after 6 weeks of high-intensity exercise. Blue symbols were the values of individual mice in the no exercise group obtained at the same time points for comparison. ** p<0.01.

**Comment 19:** Lines 284-285. Again, this statement is incorrect; your study provided NO EVIDENCE that exercise strengthened the anti-tumour efficacy of treatment because there were no sig differences. Please revise accordingly.

**Response 19: (as Comment 2&3)**

**Comment 20**: Please discuss the limitations of the study and the implications these limitations have on the findings. A major limitation is the small sample size.

**Response 20:** the limitations of the study were added into the last paragraph in the Discussion section as follows.

Line 357-364: There were several limitations in this study. First of all, the sample size was relatively small owing to the limited capacity of experiment instruments and operators. Increasing the number of animals may further strengthen the convincingness of the study. Second, we observed some changes in the blood cell components after exercise, but we had little information about the principal component which was responsible for the effects. Furthermore, the study only tested in one tumor model and should be generalized to other models such as cell lines originated from different organs, in different strain of mice, or even different species.
