# Supplementary material for: Exercise improves the outcome of anticancer treatment with ultrasound-hyperthermia-enhanced nanochemotherapy and autophagy inhibitor
Source: PLoS One. 2023 Jul 12;18(7):e0288380. doi: 10.1371/journal.pone.0288380 (PMC10337956; doi:10.1371/journal.pone.0288380)
Supplement: Attachment — Submitted filename: Response to Reviewers.docx [file pone.0288380.s003.docx]

Prof. E. Chenette

Editor-in-Chief, PLOS ONE

Dear Prof. Chenette,

The manuscript has been changed and modified point by point according to the comments and suggestions from the Reviewers. All authors have read and approved for submission of this manuscript to *PLOS ONE,* and any portion of the manuscript has not been appeared nor under consideration to other journals. We believe this combinatorial strategy significantly advances the knowledge for cancer treatment. The following are the responses for the comments point by point:

**Reviewer 1’s comment:**

**Comment:** Thank you for addressing my comments, I am satisfied with the revisions.

**Response:** We greatly appreciated your precious comments that help us improve the quality of our paper.

**Reviewer 2’s comments:**

The manuscript is well written but needs some revision before final decision.

**Comment 1:** Please revise the English language.

**Response 1:** The language usage in the manuscript was thoroughly revised.

**Comment 2:** The 4T1 breast model is a metastatic cancer model and I extremely recommend the authors investigate the effect of the recommended treatment on metastasis of 4T1 breast cancer in vital organs.

**Response 2:** Due to the time restriction of revision, we were unable to investigate the actual effects of proposed treatment on the metastases of 4T1 in vital organs. Alternatively, we reviewed literatures that addressed the interactions between exercise treatment and metastasis and added a paragraph in the Discussion section. For the coherence of the article, the prior paragraph was also revised. The revised paragraphs are as follows.

Line 352-396:

Exercise is widely recognized as a beneficial factor that reduces the incidence of cancer and improves the outcome of cancer patients from the epidemiological observations [20-24]. Nevertheless, the relation between exercise and cancer became less conclusive among preclinical studies exploring the underlying mechanisms or investigating experimental therapies with animal models [63-65]. In a systematic review collecting 53 preclinical studies about exercise therapy in cancer, exercise reduced the incidence of cancer in 58% studies and inhibited tumor growth in 64% studies [63]. However, some studies showed no benefit or even the contrary: exercise increased cancer incidence in 8% studies and accelerated tumor growth in 9% studies [63]. In another systematic review analyzing 28 preclinical studies, similar discrepancy also existed: 5 among 20 studies reported adverse effect of exercise on tumor incidence, 2 among 14 studies reported negative impact of exercise on tumor multiplicity, and 3 among 11 studies reported increased tumor volumes by exercise [64]. The inconsistency reflects the complex interactions between exercise physiology and cancer biology, as well as great heterogeneity in experimental protocols between studies. It demands more standardized protocols including exercise treatment, disease model, and evaluation metrics to generate reproducible data and consistent conclusions, such as the Exercise as Cancer Treatment (EXACT) framework proposed by Courneya and Booth [66].

The relationship between exercise therapy and metastasis is more difficult to assess due to the limited number of preclinical studies available and the notable discrepancies observed between these studies. Sheinboim et al. found that exercise prior to tumor implantation significantly protected against metastases in distant organs in three models of melanoma [67]. The protective effects of exercise were dependent on mTOR activity and was reversed by the inhibition of the mTOR pathway with rapamycin [67]. Alvarado et al. also found in an MNU-induced model that exercised Sprague-Dawley rats developed less tumors than sedentary ones and did not develop any metastasis [68]. In addition to preventing the formation of metastasis, Vojdani et al. also showed that exercise after tumor inoculation reduced the infiltration of metastatic 4T1 cells into liver and lung [69]. On the other hand, several studies indicated that exercise had no effect on metastasis. Yan and Demars reported that voluntary running did not reduce the number and size of pulmonary metastases as compared to sedentary control, either in experimental metastasis of B16BL/6 melanoma or spontaneous metastasis of Lewis lung carcinoma (LLC) model [70]. Similarly, Wakefield et al. concluded that exercise did not significantly affect the number of pulmonary metastasis of 4T1 cells [71]. It is noteworthy that the researchers also examined the impact of intraductal 4T1 tumor growth on exercise performance. As the tumors grew, the running behavior during the plateau phase was affected, and the running distance decreased [71]. There were even some studies claimed that exercise might have adverse effects on metastasis. MacNeil and Hoffman-Goetz reported that the tumor incidence was not different across activity groups, but the tumor multiplicity was higher in mice that had been previously exposed to exercise [72]. In a later study conducted by Smeda et al., they concluded that exercise with wheel running did not affect the volume of the primary 4T1 tumor, but the number of pulmonary metastatic nodules was significantly increased compared to the sedentary controls [73]. Standardized protocols and reporting system are required to eliminate the discrepancies between studies and reveal the true effect of exercise on metastasis.

**Comment 3:** The charts and figures are not prepared professionally. Please revise them in their appearance.

**Response 3:** Fig. 5, Fig. 6, and Fig. 7 (old Fig. 4-6) were re-drawn as slopegraphs to present the findings more clearly, and the figure captions were also revised accordingly. The revised figures and figure captions were as follows.

**Fig 5. The percentage of neutrophils among white blood cells (NEUT%).**

The green dotted lines indicated the changes in NEUT% of individual mice in the exercise group obtained before and after 6 weeks of high-intensity exercise, and the green solid line indicated their mean value. The blue dotted lines were the changes in NEUT% of individual mice in the no exercise group before and after the duration of 6 weeks, and the blue solid line indicated their mean value. *: p<0.05. ns: not significant.

**Fig 6. The percentage of lymphocytes among white blood cells (LYMPH%).**

The green dotted lines indicated the changes in LYMPH% of individual mice in the exercise group obtained before and after 6 weeks of high-intensity exercise, and the green solid line indicated their mean value. The blue dotted lines were the changes in LYMPH% of individual mice in the no exercise group before and after the duration of 6 weeks, and the blue solid line indicated their mean value. *: p<0.05. ns: not significant.

**Fig 7. The number of reticulocytes and the percentage among red blood cells.**

(a) The number of reticulocytes (RET) and (b) the percentage among red blood cells (RET%). The green dotted lines indicated the changes of individual mice in the exercise group obtained before and after 6 weeks of high-intensity exercise, and the green solid line indicated their mean value. The blue dotted lines were the changes of individual mice in the no exercise group before and after the duration of 6 weeks, and the blue solid line indicated their mean value. **: p<0.01. ns: not significant.

**Comment 4:** Every medical intervention has its own side effects and implications please mention yours and evaluate the side effects of your treatment in vivo.

**Response 4:** The side effects of proposed treatments were evaluated by regularly monitoring the changes in the body weights of mice. The changes in the body weights of mice was shown in a newly added figure, Fig.4, and the results were also added as a subsection in the Results section, as follows.

Line 287-293:

**Body weight changes due to treatments**

To see if the treatments caused any side effects to mice, the body weights of the mice were regularly measured every 7 days since D7 after tumor implantation. The changes in the body weights were shown in Fig 4. There was no substantial drop in body weight seen during the 60-day observation period. It implied that both treatments, HIIT+PLD+pUH+CQ and PLD+pUH+CQ, were well tolerated without obvious side effects.

**Fig 4. Body weight changes during the observation period.**

**Comment 5:** Please mention the further steps for your treatment. What is its future? which steps are needed? What are the limitations? etc.

**Response 5:** The translation from preclinical study to clinical practice in the future, along with the issues it may face in the process were discussed and added into the Discussion section, as follows.

Line 409-422:

Before the proposed combinatorial therapy can be translated from preclinical research to clinical practice, several issues must be addressed. Despite the rapid growing in the field of exercise oncology, only a small proportion of cancer patients participates in exercise interventions as a means to fight against cancer [77]. The barrier may result from the lack of knowledge, physical factors caused by disease or treatment, or psychological factors. Exercise interventions is generally considered safe and is recommended for cancer patients [78]. However, in some circumstances such as bone metastases or peripheral neuropathy, it requires extra caution to prescribe a safe and effective exercise program [78]. These special conditions do not preclude patients from exercise interventions, but the exercise program should be tailored by professionals who are familiar with the specific considerations of cancer and anti-cancer treatment [78]. Compliance is another problem. A considerable proportion of patients discontinued their exercise intervention programs prematurely due to various reasons such as disease progression or lack of motivation [79, 80].
